# Minimally Invasive Far-Lateral Microdiscectomy: A New Retractor for Far-Lateral Lumbar Disc Surgery

**DOI:** 10.7759/cureus.12625

**Published:** 2021-01-11

**Authors:** Ülkün Ünsal, Salim Senturk

**Affiliations:** 1 Neurosurgery, Koç University Hospital, Istanbul, TUR; 2 Neurosurgery, Memorial Bahçelievler Hospital, Istanbul, TUR

**Keywords:** far-lateral disc herniation, microdiscectomy, minimally invasive spine surgery, new retractor

## Abstract

Background

To date, a number of approaches have been described for far-lateral disc surgery, including midline, paramedian, and intertransverse approaches. These approaches pose challenges for surgeons due to the difficulty in retraction caused by the anatomy of the foramen. We designed a retractor suitable for the three-dimensional anatomical structure of the foramen. In this study, we aimed to evaluate the surgical outcomes of the patients who were operated on using this retractor in our clinic.

Methods

The retrospective study included patients who were operated on due to far-lateral disc herniation using the retractor designed in our clinic between February 2013 and December 2018.

Results

The study included 11 (64.7%) women and 6 (35.3%) men, with a mean age of 56 years (range: 42-70 years). The mean operative time was 49 minutes (range: 40-70 minutes), the mean estimated blood loss was 42 mL (range: 25-60 mL), and the mean follow-up period was 22.6 months (range: 13-48 months). No complication occurred in any patient. A minimally invasive discectomy was performed via the paramedian approach in each patient. The patients were evaluated using the visual analog scale (VAS) for radicular pain, Oswestry Disability Index (ODI), 36-Item Short Form Survey (SF-36), and the modified MacNab criteria.

Conclusion

The retractor developed in our study provided numerous benefits during the surgical procedure as it led to minimal blood loss and reduced operative times by avoiding bone resection in extraforaminal discs and requiring minimal bone resection in foraminal discs.

## Introduction

Far-lateral disc herniation accounts for 0.7-1.7% of all disc herniations and approximately 7% of lumbar disc herniations [1,2]. Far-lateral disc herniation often occurs at the L4-5 level [3]. Most of the disc herniations protrude into the spinal canal, thereby resulting in transverse root compression [4]. In far lateral disc herniations, however, the protruded, extruded, or sequestrated discs cause foraminal stenosis, thus leading to compression of the root and symptoms associated with the root. The radicular symptoms resulting from far lateral disc herniation are characterized by severe pain accompanied by motor deficits with or without sensorial deficits. Surgery becomes mandatory when these symptoms become apparent [5]. To date, a number of approaches have been described for far-lateral disc surgery including midline, paramedian, and intertransverse approaches [6-9]. The paramedian approach, despite protecting the medial muscle tissue, poses challenges for surgeons due to the difficulty in retraction caused by the anatomy of the foramen, thus resulting in larger incisions and ultimately leading to increased operative time and blood loss [10]. To overcome these problems, we designed a retractor suitable for the three-dimensional (3D) anatomical structure of the foramen and aimed to evaluate the surgical outcomes of the patients who were operated on using this retractor in our clinic.

## Materials and methods

**Figure 1:**
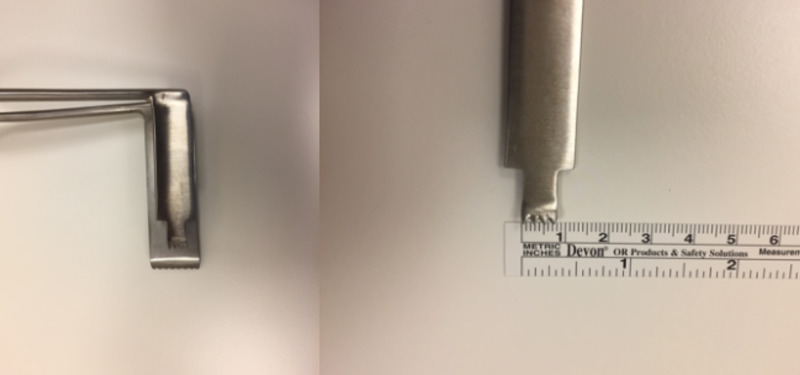
Retractor (drawing by the patent team).

This retrospective study included patients who were operated on due to far-lateral disc herniation using the retractor designed in our clinic (Figure 1) between February 2013 and December 2018. A minimally invasive discectomy was performed via the paramedian approach in each patient. The study included a total of 17 patients who had failed to respond to prior conservative treatments (non-steroidal anti-inflammatory treatment, bed rest, selective root injection, and physical therapy) and had sciatica and significant motor deficits. Only two patients had lower extremity weakness on neurologic examination preoperatively, both having weakness of ankle dorsiflexion secondary to L4-L5 disc herniation. Operative time and intraoperative estimated blood loss (EBL) were calculated for each patient. The final status of the patients was assessed by polyclinic examination. The patients were evaluated using the visual analog scale (VAS) for radicular pain, Oswestry Disability Index (ODI), 36-Item Short Form Survey (SF-36), and the Modified MacNab criteria both preoperatively and at postoperative month 12.

Structure of the retractor

**Figure 2:**
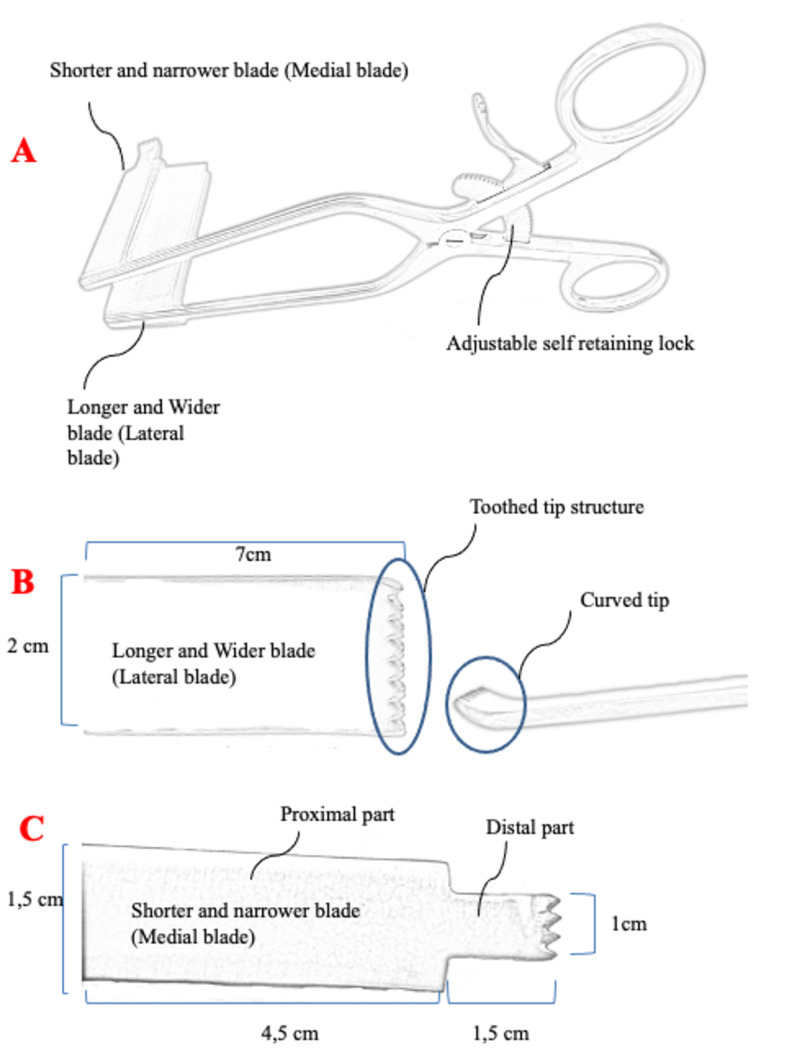
(A) Draft drawing of the retractor. (B) Lateral blade. (C) Medial blade.

The retractor was made of standard stainless steel and consisted of two parts designed in accordance with the foramen anatomy of the lumbar region. The medial blade, which was placed right above the pars interarticularis, was designed shorter and narrower (with the proximal part measuring 1.5 cm in width and 4.5 cm in length and with a shorter distal part measuring 1 cm in width and 1.5 cm in length) and the lateral blade was designed longer and wider (2 cm in width and 7 cm in length) with toothed and curved laterally in order to retract more muscle tissue (Figures 1, 2).

Surgical interventions

Operations were performed in a single center and by one surgeon who was experienced in minimally invasive surgery.

Surgical technique

**Figure 3:**
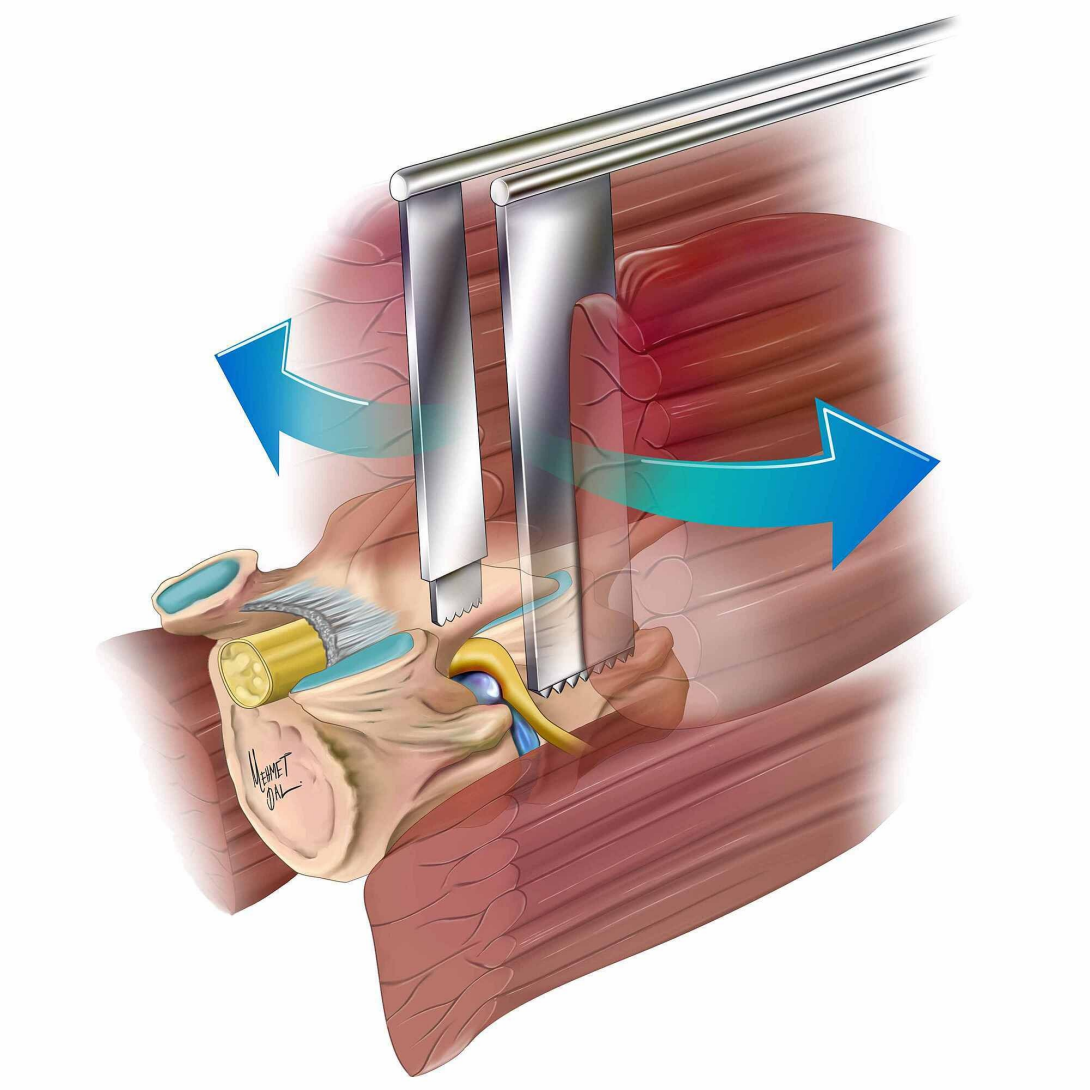
The medial (short-narrow) blade of the retractor consisted of two parts: the proximal part of the medial blade was inserted between the facet joints and the distal part was placed above the pars interarticularis. The lateral (long-wide) blade of the retractor was used to move the lateral muscles away from the surgical field.

The patient was positioned prone on a radiolucent operation table under general anesthesia. The waist of the patient was placed in the neutral position, and a pillow was placed under the abdominal region so as to lower the intra-abdominal pressure. The lateral aspect of the pars interarticularis at the level of the far-lateral disc was defined using anterior-posterior (AP) radiographs, and then the skin was marked using a skin marker. The target area was draped and prepped in a sterile fashion and then a 2.5-cm paramedian incision was made at the relevant level of the lateral border of pars interarticularis. This incision was placed by calculating the distance between the midline and the skin projection of the lateral border of the pars interarticularis after examining the axial sections of the patient’s preoperative magnetic resonance imaging (MRI) images, given that this distance may vary among patients and based on the relevant level. Fascia was dissected using electrocautery in accordance with the skin incision. The dissection was continued through the paraspinal muscle tissue until the lateral aspect of the pars interarticularis was reached. The shorter and narrower leg of the retractor was placed right above the pars interarticularis and the longer and wider leg of the retractor was inserted and opened in such a way to expose the exit of the foramen. Due to the advantageous design of the retractor and its wide and deep spoon, the lateral muscles were moved away from the surgical field (Figure 3). Meanwhile, the table was inclined toward the opposite side. As it is usually unnecessary, no bony structure was removed in extraforaminal discs. In foraminal discs, however, the superior facet of the corresponding distance was bitten by a Kerrison rongeur to remove the bone pinch by pinch. The pars interarticularis was left intact, unless necessary. If needed, less than one-fourth of the pars interarticularis was excised. The intertransverse ligament was removed or dissected, and the fatty tissue was exposed. Some portion of the soft tissue was removed by palpating the lower pedicle with a long-ended hook in order to secure safe borders and to avoid harm to the root. The disc was exposed and then the root became visible. The protruded, extruded, or sequestrated compartments of the disc compressing the root were removed. The procedure was completed after closing the fascia and then the skin.

Postoperative management

Patients were mobilized on the same day at postoperative hour 6. The patients were provided analgesics as needed. All patients were discharged one day after surgery.

Statistical analysis was performed using SPSS for Windows Version 21 (IBM Corp., Armonk, NY, USA). McNemar’s chi-square test was used to determine the differences between pre- and postoperative measurements. Normal distribution of data was determined using the Shapiro-Wilk test, and normality assumptions were rejected for each component of SF-36, including its subgroups. Differences between pre- and postoperative SF-36 scores were determined using the Wilcoxon signed-rank test. A p-value of <0.05 was considered significant.

## Results

Patient characteristics

The study included 11 (64.7%) women and 6 (35.3%) men with a mean age of 56 years (range: 42-70 years) (Table 1). The most common intervertebral level involved in our patients was L4-L5 (n=8) followed by L5-S1 (n=6), L3-L4 (n=1), L2-L3 (n=1), and L1-L2 (n=1). The mean operative time was 49 minutes (range: 40-70 minutes), mean EBL was 42 mL (range: 25-60 mL), and mean follow-up period was 22.6 months (range: 13-48 months) (Table 2).

**Table 1:** Patient characteristics EBL, estimated blood loss; FU, follow-up; F, foraminal; EF, extraforaminal

Patient	Age/sex	Level	Operating time (minutes)	EBL (mL)	FU (months)
1	46/F	Left L5-S1-F	60	55	13
2	70/F	Left L1-2-F	45	35	38
3	50/F	Right L5-S1-EF	50	40	48
4	43/F	Left L4-5-EF	40	30	15
5	60/M	Left L3-4-EF	55	45	20
6	62/F	Right L2-3-F	45	45	28
7	61/F	Right L5-S1-EF	70	60	16
8	56/M	Right L5-S1-EF	50	40	20
9	60/M	Left L5-S1-F	50	55	18
10	61/M	Right L4-5-EF	45	30	33
11	62/F	Right L4-5-EF	40	40	22
12	54/F	Left L5-S1-F	55	50	17
13	42/F	Right L4-5-F	45	30	26
14	68/M	Left L4-5-F	50	45	18
15	50/M	Left L4-5-EF	45	40	20
16	54/F	Right L4-5-EF	50	25	15
17	66/F	Right L4-5-EF	45	50	18

**Table 2:** Age, blood loss, and operative time

	N	Minimum	Maximum	Mean	SD
Age (years)	17	42	70	56.76	8.40
Blood loss (mL)	17	25	60	42.06	10.01
Operative time (minutes)	17	40	70	49.41	7.48

Follow-up and outcomes

Partial recovery was observed in one of the two patients with preoperative motor deficit, while the deficit of the other patient continued postoperatively and these patients were consulted to the Physical Therapy Department. There were no complications. No sign of recurrence was detected in any patient at 12-month follow-up. At the same follow-up visit, the patients were evaluated using VAS for radicular pain, ODI, SF-36, and the Modified MacNab criteria (Tables 3, 4).

**Table 3:** Postoperative MacNab scores p=0.0065 (McNemar)

Postoperative scores	
Excellent	9
Good	8
Fair	0
Poor	0

**Table 4:** Comparison of SF-36, VAS, and ODI test results (Wilcoxon signed-rank test) VAS, visual analog scale; ODI, Oswestry disability index; SF-36, 36-Item Short Form Health Survey

Group		Mean	N	SD	p-Value
Pair 1	Physical function - preoperative	50.29	17.00	14.08	<0.001
	Physical function - postoperative	80.00	17.00	10.61	
Pair 2	Role physical - preoperative	30.88	17.00	46.38	0.002
	Role physical - postoperative	79.41	17.00	26.86	
Pair 3	Role emotional - preoperative	47.06	17.00	37.38	0.004
	Role emotional - postoperative	90.20	17.00	28.30	
Pair 4	Vitality - preoperative	34.12	17.00	5.93	<0.001
	Vitality - postoperative	58.53	17.00	12.34	
Pair 5	Mental health - preoperative	58.59	17.00	6.62	0.004
	Mental health - postoperative	71.76	17.00	9.85	
Pair 6	Social function - preoperative	52.94	17.00	12.13	<0.001
	Social function - postoperative	76.47	17.00	10.72	
Pair 7	Bodily pain - preoperative	22.06	17.00	12.48	<0.001
	Bodily pain - postoperative	78.97	17.00	14.42	
Pair 8	General health - preoperative	49.41	17.00	9.98	0.072
	General health - postoperative	58.24	17.00	17.58	
Pair 9	VAS - preoperative	8.24	17.00	1.03	<0.001
	VAS - postoperative	2.35	17.00	0.70	
Pair 10	ODI - preoperative	78.24	17.00	7.96	<0.001
	ODI - postoperative	21.41	17.00	5.99	

Visual analog scale

Mean VAS score for radicular pain showed significant improvement from 8.24 preoperatively to 2.35 at 12 months postoperatively (p<0.001), suggesting that the radicular pain decreased significantly in all patients.

Short-form 36

Mean bodily pain score and mean physical function score improved from 22.06 and 30.88 preoperatively to 78.97 and 79.41 at 12 months postoperatively, respectively.

Oswestry disability index

The mean ODI score showed significant improvement from 78.24 preoperatively to 21.41 at 12 months postoperatively (p<0.001).

Modified MacNab criteria

According to the modified MacNab criteria, nine (52.9%) patients had excellent outcomes and eight (47.1%) had good outcomes.

## Discussion

Clinical characterization of far-lateral disc herniation was first identified in 1974 by Abdullah et al. [6]. As in other lumbar disc herniations, the first choice in far lateral disc herniation is non-operative treatment [11]. However, for patients showing no response to these treatments, surgical treatment becomes mandatory. Common surgical procedures used for far-lateral disc herniation include conventional midline approach and partial or complete resection of the pars interarticularis and/or inferior facet, which may ultimately require lumbar fusion due to the risk of instability [11-13].

Another approach to far-lateral disc herniation is the paramedian approach that provides a number of advantages such as directly targeting the affected area, preserving the facet joint, and continuing the dissection through muscle tissues without requiring bone resection in extraforaminal discs and requiring minimal bone resection in foraminal discs. Paramedian approaches provide protection against the complications associated with median approaches such as dural injury, residual sequestrated disc, and cerebrospinal fluid fistula [5]. Nevertheless, the exposure enabled by the retractors used in paramedian approaches pose a challenge for surgeons and may require a larger incision and extensive muscle dissection, possibly leading to greater blood loss and increased postoperative pain [10,14]. Conventional retractors used in paramedian approaches include Gelpi retractors [15], Caspar retractors, Williams retractors, Speculum retractors, and tubular retractors [10,16-19].

Facet joint and pars interarticularis are more commonly preserved by the Wiltse method (paramedian approach). Wiltse and Spencer described a technique using the Gelpi retractor, in which the medial tip of the retractor was removed to prevent the risk of dural injury by the insertion of both vertical tips from the facet joint to the spinal canal [15,20].

Tessitore and de Tribolet reported that the primary target in microsurgical transmuscular approach was the pars interarticularis, and they inserted a Caspar-type retractor between the muscles. The authors indicated that although that technique had numerous advantages, it had a high risk of injuring the dorsal ramus of the spinal nerve, lumbar artery, and the associated veins due to exposure [16].

Previously it was reported that the unilateral Williams retractor, which is used in paramedian or central lumbar disc herniation, is not very suitable in far-lateral lumbar disc surgery, as the medial blade of the retractor is thin and is not always able to provide adequate exposure [17].

In contrast, the medial blade of the retractor introduced in our study consisted of two parts: the proximal part of the medial blade was inserted between the facet joints, and the distal part was placed above the pars interarticularis. The lateral blade retracted the lateral muscle groups and provided adequate exposure of the foramen while preventing the risk of injuring the dorsal ramus of the spinal nerve.

Surgical approaches to L5-S1 far-lateral disc herniations still pose technical challenges despite the use of tubular retractors. In a previous retrospective study, Stavrinou et al. evaluated 11 patients who underwent far-lateral disc surgery with extraforaminal decompression of the L5 nerve root using a tubular retractor and reported that the retractor restricted the surgeons’ exposure and the possibility to become familiar with the extraforaminal decompression of the L5 spinal nerve [18]. In contrast, the retractor used in our study provided better retraction in L5-S1 far-lateral disc herniations compared to classic retractors, although the iliac crest was a problem for our patients, and there were only three patients who underwent the surgery at the L5-S1 level.

Currently, far-lateral discs can easily be operated with endoscopic techniques [21]. However, due to the high costs of the equipment used in endoscopic surgery, the long learning curve of the surgeon, and the lack of popularity of endoscopic surgery, other classical techniques are still being used extensively.

Our clinical findings regarding radicular pain and neurological and functional status were consistent with those reported in the literature. Voyadzis et al. performed a paramedian approach using tubular retractors in 20 patients with far-lateral disc herniation and obtained excellent outcomes in 60% and good outcomes in 40% of the patients according to MacNab criteria [10]. The mean operative time was 82 minutes, and the mean EBL was reported as 31 mL. In our study, similar outcomes were obtained based on the MacNab criteria, while the mean EBL was 42 mL. Epimenio et al. administered a far-lateral microsurgical approach for pure extraforaminal herniation without bone resection at a location 3-4 cm lateral to the pars interarticularis [22]. The patients were discharged and returned to daily life activities two days after surgery. Similarly, we also administered a far-lateral approach for extraforaminal discs, and the patients were discharged within two days after surgery. Salame and Lidar evaluated a series of 31 patients who underwent minimal invasive far-lateral discectomy using a tubular dilatator and reported that significant improvement was observed between pre- and postoperative mean VAS and SF-36 scores, whereby the mean VAS score for radicular pain decreased from 8.6 preoperatively to 0.6 postoperatively, the SF-36 mean bodily pain index improved from 6.71 to 79.53 postoperatively, and the SF-36 mean physical function score improved from 9.68 to 76.33 postoperatively [5]. The authors also noted that the mean operative period was 44 minutes, and the mean follow-up period was 25.16 months. In our study, the mean VAS score decreased from 8.24 preoperatively to 2.35 postoperatively, the SF-36 mean bodily pain index improved from 22.06 to 78.97, and the SF-36 mean physical function score improved from 30.88 to 79.41. Moreover, the mean operative time was 49 minutes, and the mean follow-up period was 22.6 months.

We designed a retractor suitable for the 3D anatomical structure of the lumbar foramen and employed it for the operative treatment of our patients. It is commonly known that the oval structure at the bottom of classic retractors or at the tip of the tubular retractors and nasal speculum poses challenges for surgeons as it is not suitable for the structure of the foramen. Additionally, these retractors have been shown to provide poor visibility and inadequate decompression [10,14,23-26]. On the other hand, classic retractors typically remain above the facet, and thus the tissues that penetrate into the disc through the spaces at the bottom of the disc lead to poor visibility, and as a result a wider incision becomes mandatory to provide better visibility. The retractor designed in our study eliminated these problems and provided remarkable benefits during the surgical procedure. Moreover, it led to no injury to the dorsal ramus since its medial shorter blade was suitable for the anatomy of the foramen and could be placed medially on the pars interarticularis.

Despite the advantages, there are some limitations of this study. Although the number of samples was similar to the other far lateral discectomy series, the sample size was relatively small [4,5,9,10]. The other limitation was the retrospective design of the study.

## Conclusions

The retractor developed in our study provided numerous benefits during the surgical procedure as it led to minimal blood loss and reduced operative times by avoiding bone resection in extraforaminal discs and by requiring minimal bone resection in foraminal discs. The retractor also allowed better surgical exposure and an increased surgical comfort for the surgeon. Moreover, the different lengths and widths of the retractor legs provided a partial solution to the retraction problem in L5-S1 foraminal/extraforaminal discs. We conclude that this retractor, which is highly suitable for the anatomy of the foramen and is highly cost-effective, should be at the disposal of every spinal surgeon.
